# Tumors after kidney transplantation: a population study

**DOI:** 10.1186/s12957-023-02892-3

**Published:** 2023-01-23

**Authors:** Giuseppe Ietto, Mattia Gritti, Giuseppe Pettinato, Giulio Carcano, Daniela Dalla Gasperina

**Affiliations:** 1 General, Emergency and Transplant Surgery Department, ASST-Sette Laghi and University of Insubria, Varese, Italy; 2 Department of General Surgery, Humanitas Clinical and Research Center, Milan, Italy; 3 Department of Medicine, Beth Israel Deaconess Medical Center, Harvard Medical School, Boston, MA 02115 USA; 4 Department of Medicine and Surgery, University of Insubria, Varese, Italy

**Keywords:** Cancer risk, Transplant, Immunosuppression, Population study

## Abstract

One of the main causes of post-transplant-associated morbidity and mortality is cancer. The aims of the project were to study the neoplastic risk within the kidney transplant population and identify the determinants of this risk. A cohort of 462 renal transplant patients from 2010 to 2020 was considered. The expected incidence rates of post-transplant cancer development in the referenced population, the standardized incidence ratios (SIR) taking the Italian population as a comparison, and the absolute risk and the attributable fraction were extrapolated from these cohorts of patients. Kidney transplant recipients had an overall cancer risk of approximately three times that of the local population (SIR 2.8). A significantly increased number of cases were observed for Kaposi’s sarcoma (KS) (SIR 195) and hematological cancers (SIR 6.8). In the first 3 years post-transplant, the risk to develop either KS or hematological cancers was four times higher than in the following years; in all cases of KS, the diagnosis was within 2 years from the transplant. Post-transplant immunosuppression represents the cause of 99% of cases of KS and 85% of cases of lymphomas, while only 39% is represented by solid tumors. Data related to the incidence, the percentages attributable to post-transplant immunosuppression, and the time of onset of neoplasms, particularly for KS and hematological tumors could help improve the management for the follow-up in these patients.

## Introduction

Kidney transplantation significantly increases life expectancy and life quality when compared to dialysis in end-stage renal disease patients (ESRD) [1–3]. However, the use of immunosuppressive drugs that are needed to prevent graft loss is directly associated with an increased frequency of infections and cancers, which are one of the main causes of morbidity and mortality in transplanted patients [4]. Nowadays, post-transplant malignancy is the third most common cause of death in renal transplant recipients, with some malignancies occurring at much higher rates compared to the general population. Prolonged exposure to immunosuppressive drugs seems to adversely affect the antitumor immune surveillance capacity and enhance the carcinogenic effect of some risk factors, such as ultraviolet rays. Furthermore, some immunosuppressive drugs appear to promote carcinogenesis independently from the immunosuppressive mechanism [5, 6].

Taking advantage of the recently acquired knowledge in carcinogenesis, the quantification of cancer risks in transplanted recipients could add an important layer when programming the follow-up screenings in these patients. We need to take into consideration that the risk of developing new cancer may vary depending on the type or location where cancer will arise.

Moreover, there is an increased risk to develop a “common” type of cancer in a transplanted patient, because the current global impact is already high per se than developing a more rare one [7].

Several studies have shown an inversely age-related risk of developing malignancies after transplantation, where younger recipients experience a much greater relative risk than older recipients (risk increased 15–30 times for children, but double for those transplanted over 65 years) [8, 9]. In addition, the risk of developing a new post-transplant cancer is estimated at about 40% for those patients who already have another tumor in their clinical history [7]. Regarding the various types of cancer, the relative increase in incidence is most significant for Kaposi’s sarcoma (KS), non-melanoma skin cancer, and lymphoma. In contrast, the risk of ovarian, prostate, and multiple myeloma cancers would not seem to be increased [10].

Post-transplant KS may arise from the reactivation of latent HHV-8 infection in endemic areas or the acquisition of new infection in non-endemic areas. An example of the first case was described by Luppi et al. in 2000, where the formation of a KS post-transplantation was due to an infected donated kidney which lead to a new acquisition of HHV-8 infection in the recipient [11]. Barozzi et al. in 2006 reported an episode of KS in a transplanted patient deriving from the reactivation of a latent HHV-8 infection of a donated kidney [12].

Not only does the risk of developing de novo cancer increases after kidney transplantation, but the prognosis for recipients diagnosed with post-transplantation cancer worsen compared to a non-transplanted patient.

Most of the tumors diagnosed in transplant recipients have more aggressive behavior, as evidenced by the Israel Penn Registry, which has shown that mean survival for certain cancers, such as colon, lung, breast, prostate, and bladder cancer, is significantly lower in transplanted patients than in the general population [7].

The Australian and New Zealand Dialysis and Transplant Registry demonstrated that transplanted women that underwent kidney transplantation and developed breast cancer have excess mortality of at least 40% compared to women with breast cancer in the general population [13–15]. In a Dutch study, the median survival of kidney transplant recipients after cancer diagnosis was only 2.7 years, compared with an average survival of 8.3 years in cancer-free recipients [16].

Another consideration that should be given attention is the phenomenon of “chimerism,” although is a concept that is still debated. In fact, in addition to “de novo” tumors, although rare, tumors “donor-transmitted” (DTT) and “donor-derived” (DDT) are clearly described in the scientific literature [17–20].

The transplant recipient is a chimera subject when two cellular populations exist. When a tumor develops shortly after transplantation, a transmission of malignancy from the donor should be considered, despite the accurate screening for the already ongoing neoplastic diseases before donation. An arbitrary 2-year cutoff time was stated to separate “donor-transmitted” from “donor-derived” tumors, the latter arising from donor cells but not present at the time of transplant. However, 31% of donor-transmitted tumors arose after 24 months, emphasizing the need for continued surveillance beyond the conventional 2 years [21].

According to the data available, the risk of having a donor with undetected malignancy is 1.3%, and the following risk of cancer transmission is 1% [17].

The “donor-derived” tumors are extremely rare. In the scientific literature, there are few cases of DDT developed outside the graft with the genome of the donor who never experienced malignancy before. Between the tumors developed in the recipients through this modality, we can find skin tumors, acute promyelocytic leukemia, Kaposi’s sarcoma, small cell carcinoma, hepatocellular carcinoma, and pancreatic adenocarcinoma [17, 22–26].

Few cases of tumors developed on the graft but originated from cells of the recipient that cannot be considered the result of a metastasization process have been described. Among them, we can find renal cell tumors developed on the renal graft several years after transplantation [27–31].

Studying the chimerism of tumors in transplant recipients could be extremely useful and should be advised in order to identify chimeric cells within these neoplasms. This identification could lead to findings on [1] the mechanism of migration of these chimeric cells into cancer and, particularly, what could be the triggering factor initiating this migration and [2] identify the cells that start the chimeric process which lead to migration of chimeric cells into the tumor [32].

In conclusion, a transplanted patient who develops a donor-derived tumor might be a useful model to distinguish between tumor cells derived from the donor and the ones of the recipient within the same organ where the tumor arose, which still show a normal phenotype. This might allow us to potentially recapitulate tumor phylogenesis.

By identifying the migrated cells from the donor organ into the recipient body, if they will give rise to a tumor, these will be recognizable as donor-derived progenitor cells by the fact that they will still possess the donor genotype. This will allow us to potentially study, through our proposed model, the hierarchy of how a tumor develops into the recipient taking advantage of the genotypical differences between donor and recipient genotypes.

## Materials and methods

This study analyzed the patients’ data transplanted at an Italian Transplant Center (ASST Sette Laghi, Varese, Italy) between January 1, 2010, and December 24, 2020.

A retrospective cohort of 462 renal transplant patients was considered.

Information was collected taking into consideration the socio-demographic aspects (sex, date of birth), the clinical history (including the presence of tumors before transplantation), and the diagnosis of tumors in the post-transplant period and current clinical conditions, from clinical records related to transplantation and post-transplant outpatient visits.

The neoplastic risk was calculated based on the reference to the first diagnosis of cancer. Cancer diagnoses were classified using the International Classification of Disease ver. 10 (ICD-10).

Statistical analysis data was performed using Microsoft Office – Excel® 2019, MedCalc® 19.5.3.

The cancer risk period (expressed in person-years (PY)) was calculated from the 30th day following the date of transplant up to the date of cancer diagnosis, death, and last follow-up or the end date of the study (March 31, 2021).

The reference population used to compare the incidence of tumors in transplanted patients was the population of the Province of Varese (Italy) of the same age and sex. The SIR, obtained by dividing the number of cases of cancers observed by the number of expected cases, was used. The number of expected cancers was obtained by multiplying the transplant recipients’ PYs at cancer risk by the sex-specific and age-specific incidence rates found in the cancer registries of the International Agency for Cancer Research (IARC, vol XI) that referred to the population of the Province of Varese. For the SIR, exact 95% confidence intervals (95% CI) were calculated according to the Poisson distribution. Estimates of the SIR and relative confidence intervals were made for the total number of patients and by gender.

The expected rates of incidence in the reference population were also calculated, and based on these, the absolute risk (AR) (i.e., the excess incidence of cancer due to kidney transplantation) and the attributable fraction (AF) were derived. The risk factors associated with the onset of cancer were also evaluated [8].

The method used to calculate a confidence interval for the difference between two proportions is the Newcombe-Wilson method without continuity correction (1998). The confidence limits for the number needed to be treated are the inverse of the limits for the AR reduction. Confidence intervals for relative risk (RR) and odds ratios (OD) are calculated using the methods described by Armitage and Berry (Armitage P and Berry G (1994): Statistical Methods in Medical Research (3rd ed.). London: Blackwell, p 131). The RR reduction and its confidence limits are one minus the relative risk and its confidence limits.

## Results

A total of 462 kidney transplant recipients [313 male (67.7%), 149 females (32.3%)], mean age at the time of transplantation of 54 ± 11 years (range 19–76), with a follow-up time up to 2562.5 person-years for the total population (1714.3 for the male, 848.3 for the female) were included in this analysis.

All patients had undergone induction therapy followed by maintenance therapy. As the standard protocol of the Transplant Center, an association of calcineurin inhibitor (CNI), antimetabolites (mycophenolate mofetil (MMF) or mycophenolic acid), steroids, and basiliximab had been used for induction therapy; thymoglobulin or plasma exchange associated with intravenous immunoglobulin had been added to the standard protocol only when required for higher immunologic risk of the recipients or for grafts from extended criteria donors.

Twenty-eight people (20 males, 8 females; 6% of the total) developed cancer (819.5 cases/100,000 PY). Table 2 shows the distribution of tumor types and the corresponding SIR. The most frequent types were non-melanoma skin cancers (7 cases), KS (5 cases), hematological tumors (5 cases), and urinary tract tumors (4 cases).

The reference population for comparing the incidence of tumors in organ transplant patients was the population of the Province of Varese (Italy) of the same age and sex, using the registries of the International Agency for Cancer Research (IARC, vol XI), so that environmental factors can be minimized.

Kidney transplant recipients showed an overall risk of cancer that was about three times (SIR 2.8; 95% CI 1.8–4.3) compared to the local population, with an overall risk for males of about double (SIR 2.3; 95% CI 1.3–4.0) and for females over triple (SIR 3.7; 95% CI 1.8–7.5). Significantly increased risks in both sexes were observed for KS (SIR 195), lymphomas, and leukemias (SIR 6.8) (Table 1).

**Table 1:** Distribution and type of neoplasms of the sample, standardized incidence ratio (SIR), and 95% confidence interval (IC95%)

Type of tumors or localization (ICD-10)	No. of subject				SIR (IC95%)					
	Total		M	F	All		Men		Women	
Kaposi sarcoma (C46)	5		2	3	195	22.8–1624				
Skin cancers (C44)	7		7	0						
Melanoma (C43)	1		0	1						
Respiratory tract (C34)	3		2	1						
Digestive tract (C17)	1		1	0						
Lymphomas/leukemias (C83–C85–C96)	5		5	0	6.8	2.6–17.7				
Urinary tract (C64–C67)	4		2	2						
Others (C50–C80)	2		1	1						
All types of cancer (C00–97)	28		20	8						
All types of cancer except non-melanoma skin (C00–97 excluding C44)	21		13	8	2.8	1.8–4.3	2.3	1.3–4.0	3.7	1.8–7.5

**Fig. 1:**
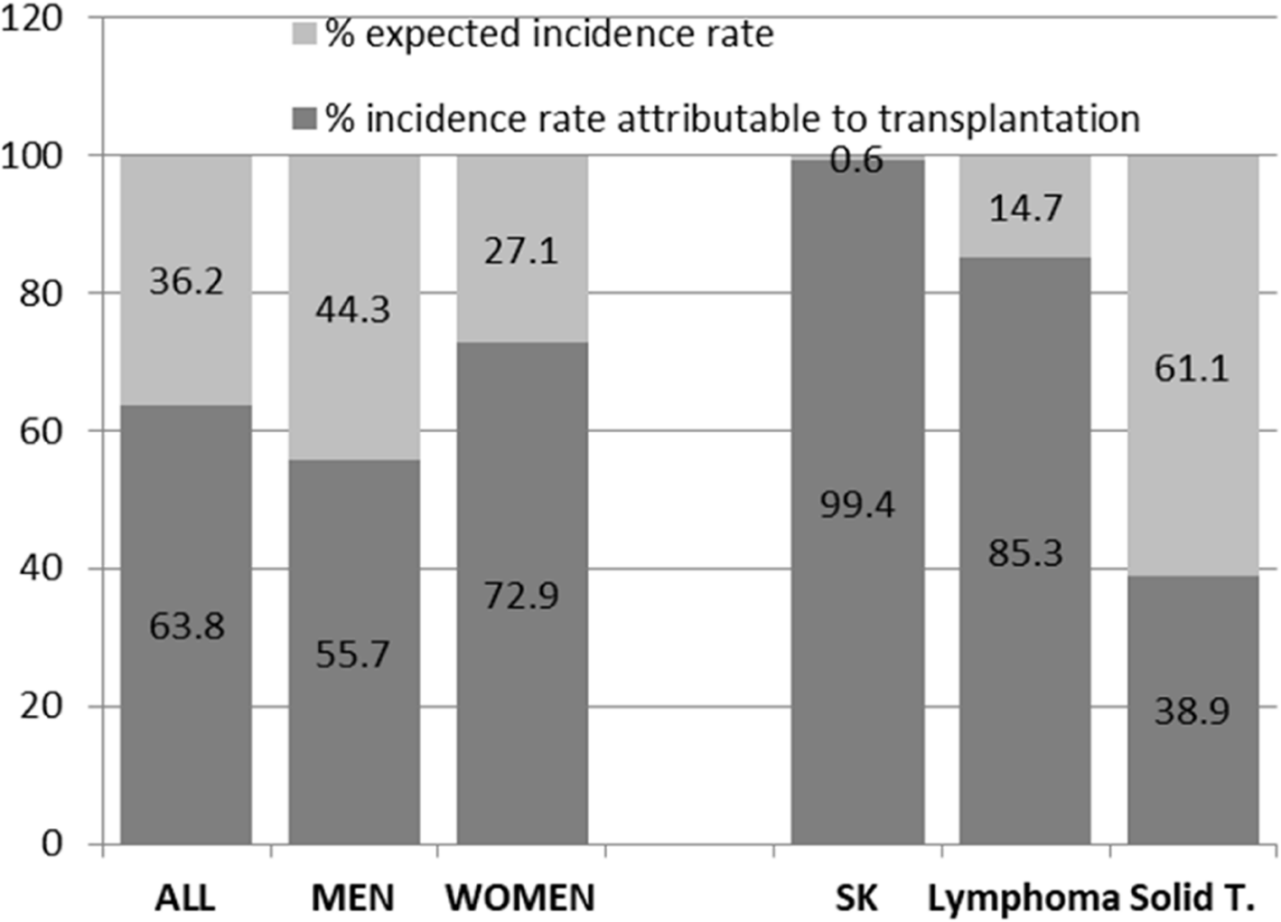
Expected and attributable incidence rates of transplantation

Overall, the incidence rate of tumors attributable to post-transplant immunosuppressive therapy was 819 cases/100,000 PY. In particular, the incidence rates for KS and lymphomas were largely attributable to post-transplant immunosuppression, while the incidence attributable to post-transplant immunosuppression for solid tumors was just over one-third of the overall incidence (162 cases/100,000 PY out of a total of 429 cases/100,000 PY) (Fig. 1).

Cancer risk increased significantly with age, while women showed a reduced risk, although not statistically significant, compared to men. Other significant associations concerned the period of tumor development after transplantation and the initiation of immunosuppressive therapy: in the first 3 years post-transplant, the risk was four times higher than in the subsequent years.

KS was diagnosed in 5 recipients at a median of 6.4 months (range 2–24) after transplant. Lesions were localized only to the skin of the extremities in 3 patients, to the skin and gastric mucosa in 1 patient, and to the inguinal lymph node in another patient. Four patients were positive at the HHV-8 serologic testing included in pre-transplant screening, while one recipient was not tested. HHV-8-DNA at KS diagnosis was detected positive in 3 recipients (Table 2).

**Table 2:** Relationship between incidence rates (IRR) and their 95% confidence intervals, for potential risk factors

	Neoplasm	
	IRR	IC95%
**Sex**		
Women	1	
Men	1.24	0.52–2.80
**Age (years)**		
< 50	1	
> 50	3.4	1.2–9.7
**Years post-transplant**		
> 3 anni	1	
< 3 anni	4.25	1.5–12

Twenty-seven people (5.8% of the total) developed a benign neoplastic form or a precancerous lesion: of these, 10 were skin lesions (3 lipomas, 2 dysplastic nevi, 2 hemangiomas, 1 neurofibroma, 1 poroma, 1 polyp fibroepithelial), 7 cellular atypia of the urinary tract with 1 bladder submucosal leiomyoma, 5 gastrointestinal lesions (4 colorectal adenomas, 1 gastric glandular polyp), 2 lung lesions (endobronchial metaplasia with dysplasia), 1 benign thyroid nodule, 1 leiomyoma ovarian, and 1 condyloma acuminata.

## Discussion

The overall increase in cancer risk found in our study (2.8 times) is in line with the published data of SIR for cancers related to kidney transplantation, taken from national population-based studies [7, 33].

Our results also estimate a 6-fold increase in the risk of hematological cancers, slightly underestimated compared to studies from Australia, Canada, and the USA, where an increased risk of developing lymphomas was related particularly to post-transplant lymphoproliferative disorders (PTLD) [34].

PTLD is one of the clinical consequences associated with EBV infection or reactivation. In fact, EBV is a ubiquitous viral pathogen, with a seroprevalence of more than 90% in adults. After primary infection, the virus persists within B lymphocytes for life with the majority of hosts demonstrating no evidence of active infection or replication. However, in kidney transplant recipients, both acute infection and reactivation of latent infection may lead to pathology, with clinical syndromes associated with non-neoplastic viral replication on one end, and EBV-mediated neoplastic transformation, including PTLD, on the other.

Transplant patients are also vulnerable to many other viral infections or the reactivation of latent infections, which can be considered one of the reasons for tumorigenesis in these subjects. Among these viruses that can cause initiation of malignancies, we can find human herpesvirus 8 (HHV-8), human papillomavirus (HPV), Merkel cell polyomavirus, hepatitis B virus (HBV), and hepatitis C virus (HCV). There is a linear correlation between the speed at which some malignant tumors develop, even after transplantation, and the initiation of immunosuppression, which could be related with uncontrolled viral replication. In support of this hypothesis, previous studies have demonstrated that recipients whose transplanted kidneys have been removed due to failure, or after reduction or cessation of immunosuppression, have lowered the risks of developing virus-induced cancers at levels observed in pre-transplant dialysis patients [35].

In regard to the development of KS, we found an increased risk in our population in agreement with the literature data, which report the risk of KS in solid organ transplant recipients at least 200- to 500-fold greater than in the local population [36–38].

The onset of KS was in all cases within 2 years after transplantation, as reported in previously published studies [38–40]. In four of our recipients, pre-transplant seropositivity for HHV-8 suggests rapid reactivation of latent infection favored by immunosuppressive therapy, particularly with calcineurin inhibitors.

Although screening for HHV-8 has not been routinely included in the pre-transplant guidelines and currently available serological tests are not optimal for variable sensitivity and specificity, the identification of high-risk patients would allow for careful post-transplant follow-up [41, 42].

In our study, cancer risks attributable to post-transplant immunosuppression are 99% for KS and 85% for lymphomas, while the fraction of solid tumors represents a small part of the observed cases in post-transplanted patients (38%). This result is believed to be related to impaired immune control of the oncogenic viruses (i.e., HHV-8), which can be present in the recipient prior to transplant or transmitted at the time of transplant via the donor organ.

A similar situation can be seen in patients with a compromised immune system, i.e., HIV/AIDS patients, where there is an increased risk of contracting cancers, while with post-transplanted patients, after immunosuppression withdrawal with the consequential return to dialysis regimen, we can observe a return to normal susceptibility to neoplastic risks.

This finding may represent useful information for post-transplant follow-up and the management of patient screening and monitoring. We need also to consider that the neoplastic risk is significantly increased in the first 3 years from the date of transplantation and, therefore, from the start of immunosuppressive therapy. An early diagnosis, for example, of KS, in which the mainstay of treatment is based on the minimization of the immunosuppression and treatment with an mTOR inhibitor, could be fundamental to treating cancer but also avoid graft rejection.

Another important aspect that needs to be taken into consideration is the study of pre-neoplastic lesions. Various pre-cancerous lesions were found in our analysis, but the recorded number could be underestimated. In immunosuppressed subjects, these lesions, such as pre-malignant polyps of the digestive cavity, tend to degenerate more rapidly into tumors than in the general population and must be diagnosed accurately during the follow-up before their malignant transformation [43].

It should be emphasized that, when comparing the neoplastic risk of the transplanted population with the one of the local population, the post-transplant path of the transplanted population should be taken into consideration.

In fact, transplant recipients are subjected to very close periodic visits by our center, considerably reducing the risk that severe complications such as tumors may not be adequately recorded, particularly in the first years of follow-up.

However, a dedicated dermatological and hematological follow-up pathway could be advisable, possibly sustained by specific guidelines. In this way, the necessary improved information of the patients concerning the post-transplant cancer risk [44], extremely helpful for the monitoring and compliance to follow-up, could be associated with the relief derived from the awareness solid strategy for the management of the risk.

The present study has limitations. First of all, the relatively small sample size and the retrospectively collected data do not permit a definitive conclusion about the neoplastic risk in the post-transplantation and do not allow to provide accurate estimates of the SIR for the low statistical power.

Due to the long period of years covered by this survey, the incidence rates from Italian cancer registries available at the time of the analysis were used.

Although this approach is frequently used, it should be noted that the intake of the rates is constant before and after the periods mentioned and cannot be satisfied for all the cancers analyzed. Likewise, they could not take into consideration the geographical differences in the incidence of tumors in the general Italian population due to poor coverage of extensive cancer registry areas.

## Conclusion

In conclusion, the results of this study validate the concept that an association between immunosuppression and cancer might exist, especially for tumors linked to viral infections.

The data related to the incidence and percentages attributable to the post-transplant immunosuppression of neoplastic diseases, such as lymphomas and KS, could improve the management of the recipients regarding the post-transplant follow-up, so that we can anticipate the tumor diagnosis, especially during the 3 years following the transplant. This might be extremely helpful because, as previously mentioned, tumors developed in transplant recipients are often more aggressive and develop at a much more advanced pace than in non-transplant patients.

## Data Availability

All complete data are available if requested.

## Abbreviations

SIR: Standardized incidence ratios
KS: Kaposi’s sarcoma
ESRD: End-stage renal disease patients
EBV: Epstein-Barr virus
HHV-8: Human herpesvirus 8
HPV: Human papillomavirus
HBV: Hepatitis B virus
HCV: Hepatitis C virus
DTT: Donor-transmitted tumor
DDT: Donor-derived tumor
ICD-10: International Classification of Disease ver. 10
IARC: International Agency for Cancer Research
CI: Confidence intervals
AR: Absolute risk
AF: Attributable fraction
RR: Relative risk
OD: Odds ratios
PTLD: Post-transplant lymphoproliferative disorders
